# Entropy analysis of MHD hybrid nanoparticles with OHAM considering viscous dissipation and thermal radiation

**DOI:** 10.1038/s41598-023-50865-z

**Published:** 2024-01-11

**Authors:** Farwa Waseem, Muhammad Sohail, Nida Ilyas, Emad Mahrous Awwad, Mohamed Sharaf, Muhammad Jahangir Khan, Ayele Tulu

**Affiliations:** 1 Department of Mathematics, Khwaja Fareed University of Engineering & Information Technology, Rahim Yar Khan, 64200 Pakistan; 2 Department of Electrical Engineering, College of Engineering, King Saud University, P.O. Box 800, 11421 Riyadh, Saudi Arabia; 3 Department of Industrial Engineering, College of Engineering, King Saud University, P.O. Box 800, 11421 Riyadh, Saudi Arabia; 4 Department of Advance Materials and Technologies, Faculty of Materials Engineering, Silesian University of Technology, 44-100 Gliwice, Poland; 5 Department of Mathematics, CNCS Ambo University, Ambo, Ethiopia

**Keywords:** Mathematics and computing, Nanoscience and technology, Physics

## Abstract

This research explores the 3-D flow characteristics, entropy generation and heat transmission behavior of nanofluids consisting of copper and titanium in water as they flow across a bidirectional apparent, while considering the influence of magneto-hydrodynamics. The thermophysical properties of nanofluids are taken advantage of utilizing the Tiwari and Das demonstrate. The concept of the boundary layer has facilitated the comprehension of the physical ideas derived from it. By applying requisite transformations, the connected intricate sets of partial differential equation have been converted into ordinary differential equation. The modified model is calculated employing the widely recognized technique known as OHAM by using Mathematica program BVPh2.0 Software. For different dimensionless parameters computational and graphical investigations have been performed. It is notice that as fluid parameters change, they exhibit distinct responses in comparison to the temperature, velocity profiles and entropy generation. The results show that velocity profile rise with greater estimates of the magnetic parameter and the rate of entropy formation. Furthermore, thermal profiles become less significant as Eckert and Prandtl numbers increase.

## Introduction

A novel kind of nanofluids known as hybrid nanofluids was created by distributing two unique nanoparticles into a common energy transport fluid. Sidik et al.^1^ compared convectional thermal transfer substances (oil, water, and ethylene glycol) and tinyfluids containing single tinyparticles. They determined hybrid nanofluids may provide greater thermal transport performance and thermophysical appearances. Yang et al.^2^ studied hybrid nanofluids as prospective fluids with well thermophysical features and thermal presentation than typical monaural-nanofluids used for thermal conveyance. Yousefi et al.^3^ studied a constant global 3-D stagnation region circulation of an aquatic titania-copper hybrid tinyliquid via a cylinder. Abdollahi et al.^4^ analyzed energy transmission to a hybrid nanofluid movement in a circling structure that contains graphene oxide and copper fragments in pristine water. They looked at how is less heat transmission when the Reynolds coefficient rises. Algehyne et al.^5^ researched heat transport in Maxwell hybrid nanofluids over an infinitely stretchy vertically permeable surface. They examined the mobility of hybrid tiny particles and discovered that when Forchheimer and Darcy's porosity constants are larger, the movement of the tinyparticles declines. Govindarajulu and Subramanyam Reddy^6^ generated magnetohydrodynamic continuous circulation of a third-grade hybrid nanoliquid in a transparent tunnel under the effects of viscous dissipation and radiant heat. Yashkun et al.^7^ observed at the motion and heat transmission of a hybrid nanoliquid via a constantly stretched/shrinking film in addition to combined convection and Joule heating. In conjunction with an innate continuous magnetic flux, Slimani et al.^8^ investigated MHD organic convection heat exchange of a hybrid tinyliquid in a conical form. They looked at how directly an electromagnetic flux passing through an open medium affects the Nusselt index. Asghar et al.^9^ inspected the impact of radiant heat on the three-dimensional magnetized rotational migration of a hybrid nanoliquid across the stretching/shrinking barrier. They found that both system thermal sketches increase when Eckert and Radiation variables increase. Upreti et al.^10^ looked into the entropy production and thermal transmission of unstable pressing magnetically hybrid tinyfluid movement among adjacent sheets by taking into account the thermal sink/source and thermal radiation. For a stationary area of a rotating sphere including chemical reactions, Nasir et al.^11^ investigated the pair stress Casson hybrid nanofluid using time-varying MHD quadratic thermal transport mobility. We conclude that in this article authors do not use the heat generation term. On a smooth, unstable stretchy paper, Nasir et al.^12^ examined the Darcy Forchheimer 2-dimensional tiny film fluids of tinyliquid.

Here, paired uncertain differential equalities (DEs) are governed by the Optimal Homotopy Analysis Technique (OHAT). Biswal et al.^13^ utilized the convergent controller settings in OHAT to reduce the difficulty of the computations. Rasool et al.^14^ employed the Optimal Homotopy Analysis Methodology (OHAM) for the investigation of the characteristics of the Cattaneo-Christov system in sticky and chemically explosive tiny fluid motion. After introducing resemblance components to the governing movement calculations, Prasad et al.^15^ used the optimum homotopy analysis approach to obtain the results for the differential equalities structure. Abolbashari et al.^16^ employed the Optimum Homotopy Analysis Model (OHAM) to determine the thermal conductance of naturally convective increased entropy generation. Obalalu et al.^17^ evaluated the impact of thermophoresis and Brownian mobility using the Optimum Homotopy Analysis Model (OHAM) strategy. For the associated governing differential of the algorithm, Biswal and Chakraverty^18^ utilised the Optimized Homotopy Analytic Model (OHAM) to obtain an approximatively series approach. To estimate the convergent controller settings utilized in OHAM, they suggested a novel technique. In this study, Wang et al.^19^ examined the properties of thermal transfer in a 2-D extended viscoelastic nanofluid flowing in a steady 3-D flow. Utilizing Cattaneo-Christov concept in conjunction with Buongiorno's model, a new model for heat conduction is established. A negative correlation has been found between the number of thermal relaxation time constraints and the thermal energy transportation. Mabood^20^ contrasted the homotopy perturbation approach with the optimum homotopy asymptotic method for severely nonlinear equations. They discovered that the Optimum Homotopy Analysis Approach and the numerically atypical Homotopy Perturbation Methodology are in good agreement for all parameter quantities. Alnahdi et al.^21^ evaluated a set of non-dimensional border value problems may be analytically solved using the homotopy analyses approach.

In radiation, pulses are released or transmitted from outside nanoparticles or across space in the way of heat. Additionally, radiation affects a fluid's speed of heat dispersion and other thermo-physical characteristics. Nayak^22^ a conducted three dimensional magnetohydrodynamic (MHD) movement and heat transmission study related to heat radiation and viscous dissipation of tiny fluid. In accordance with the temperature profile, Madaki et al.^23^ detected heat production and absorbance. Zainal et al.^24^ explored the motion and heat transport properties of a hybrid tinyfluid (Cu–Al_2_O_3_/water) in the existing of magnetohydrodynamics and radiant heat. Mabood et al.^25^ studied the immutability of the Fe_3_O_4_–Co/kerosene hybrid tinyfluid through a wedge with irregular radiation and a heat source. Hayat et al.^26^ observed the impacts of radiant heat and an applied magnetic field on Jeffery fluid with peristalsis. They examined the oscillatory behavior of the heat transmission coefficient to enable more accurate calculations of the radiation factor. Nasir et al.^27^ examined the effects of nanomaterials as well as warmth exchange occurrences exposed to thermal radiation. The circulation during thermal radiation and electro-magnetohydrodynamics was studied by Nasir et al.^28^ using a time-independent detachable stagnant spot on a Riga sheet. In regard to transferring flow, Gul et al.^29^ investigated the hybrid tinyfluid thermal using a variety of hybrid nanofluid fractional factors. Nasir et al.^30^ examined the key features of the Lorentz effect as a function of the magnetic field and the power and mass transfer mechanism with nonlinear heat radiation coupled with hybrid nanoliquid. Through a 3D stretchy exterior, Nasir and Berrouk^31^ investigated the complexities of mixed convectional boundary coating movement and magnetohydrodynamic border layer movement with regard to coupling stress Casson tinyfluid mechanics. The thermal energy transfer assessment with chemotaxis in the spontaneous laminar flow of viscous nanofluid across extensible vertically slanted heated surface was investigated by Wang et al.^32^. It is observed that the spontaneous laminar flow field rises close to the sheet's surface before exponentially decaying to the free stream. Nasir et al.^33^ examined a dynamic MHD Darcy-Forchheimer fluid movement with radiative impact over an indefinitely porous extended medium. Nasir et al.^34^ explored the effect of nonlinear radiant energy on the magnetic hydrodynamics (MHD) motion of a pair stressed water-soluble nanotechnology, hybrid, and tripartite hybrid nanofluids on a stretched surface. When various kinds of fluids undergo thermal alterations, heat is produced. These substances are known as heat-generating liquids. This occurrence has a negative impact on coolant heat exchange. Wang et al.^35^ investigated an asymmetry system combining the features of biologic convection into a stretched Maxwell nanofluid (MNF) employing Fourier and Fick model in a symmetrical extensible slipping. It has been determined that the heat profile is enhanced at higher temperatures and concentrations while using Fourier setups to figure out the present Biot amount and basic MNF factor. Sivasankaran et al.^36^ used a regional warmth chaotic simulation to analyze the convective motion and heat transmission of tiny fluids in an inclination chamber composed of heat-generating permeable media. In the manifestation of the heat generating effect, Mansour et al.^37^ examined the regular convective thermal exchange in an inverted triangle enclosure containing Cu-water tiny fluid saturated spongy media. They investigated the relationship between a rise in the thermal production index and a fall in the median Nusselt quantity. Selimefendigil et al.^38^ investigated varied convection in a chamber with volumetric heat production, containing tiny fluid and internal spinning tube. Mamun et al.^39^ induced the influence of producing heat on regular convective motion and conduction within a vertical smooth platter. They looked at whether the fluid temperature rose as the thermal production ratio rose. Shivakumara et al.^40^ examined the effect of overflow and inner thermal production on the beginning of convection in an unending horizontal liquid level. Nasir et al.^41^ studied Magnetised sectors, nonuniform heat production, dispersion, Ohmic warmth, Darcy-Forchheimer porosity area, and chemical interactions with activating energies into the transmission dispersion. Wang et al.^42^ conducted an investigation on the transport characteristics of heat and mass in a 3-D flow of Maxwell nanoliquid through a stretched superficial via a porous media. The unique properties of Maxwell nanofluids have garnered considerable research interest and attention, underscoring their broad potential applications across various fields.

The recognized Das and Tiwari nanofluid model is used in this investigation. In this investigation, numerous form parameters are taken into account together with the impact of magneto-hydrodynamics. Our research presents a terminology for defining the dispersion of Cu and TiO_2_ nanoparticles in a base fluid, specifically water. This formulation incorporates a model that takes into consideration the influence of a magnetic field and the Joule heating occurrence. In addition, we examined the various kinds of nanoparticles listed in Table 2. The structure of the study is as follows: the introduction is in “Introduction”, and the model aspects and numerical modelling are in “Model features and numerical modeling”. The method of solution is examined in “Method for optimal homotopy analysis”, and the physical implications of the solution are covered in “Explanations regarding graphical outcomes” section. Section 5 presents the key findings which are covered in “Conclusion” section.

## Model features and numerical modeling

The basic considerations are listed below.

**Figure 1:**
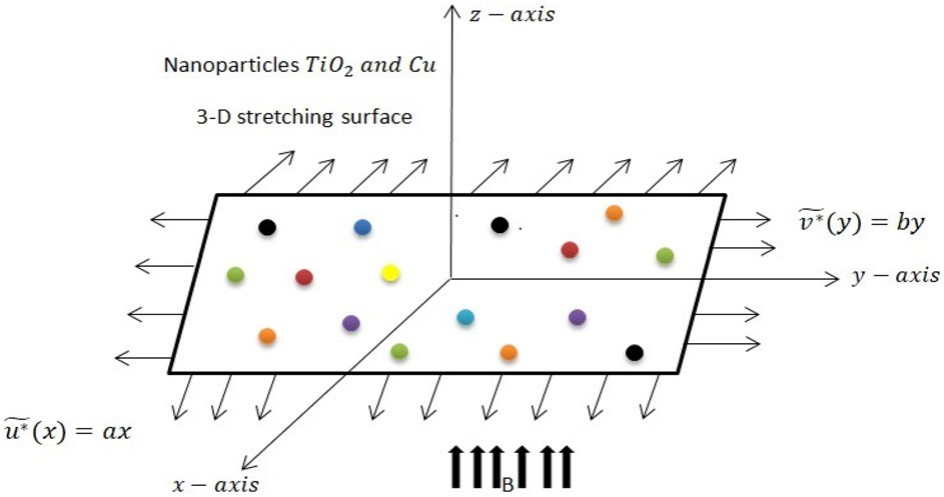
In terms of order, the geometry of the fluid flow phenomenon.

- TiO_2_–Cu/water nanofluid flow with an incompressible boundary in 3 dimensions.
- The condition where the surface moves at velocities of $\widetilde{{u}^{*}}={U}_{w}\left(x\right)=ax$ and $\widetilde{{v}^{*}}={V}_{w}\left(x\right)=by$ while the origin stays stationary will be examined in this study. In this case, the positive parameters $a$ and $b$ signify stretching in both the $x$ and $y$ directions.
- The extendable surface is subjected to a magnetic field $B$ that is directed perpendicularly.
- Numerous shapes of nanoparticles including brick, sphere, cylinder, blade, and platelet, were also examined.
- Thermal radiation, magnetic dipole, and heat generation/absorption are considered.
- Water is regarded as base fluid.
- The geometry of the model is depicted in Fig. 1.

According to the model stress tensor is defined as

1 $$\widetilde{{\tau }^{*}}=-PI+\mu {\mathbb{A}},$$

2 $${\mathbb{A}}={\mathbb{L}}+{\mathbb{L}}^{t}.$$

where

3 $${\mathbb{L}}=\left[\begin{array}{ccc}\frac{\partial \widetilde{{u}^{*}}}{\partial x}& \frac{\partial \widetilde{{u}^{*}}}{\partial y}& \frac{\partial \widetilde{{u}^{*}}}{\partial z}\\ \frac{\partial \widetilde{{v}^{*}}}{\partial x}& \frac{\partial \widetilde{{v}^{*}}}{\partial y}& \frac{\partial \widetilde{{v}^{*}}}{\partial z}\\ \frac{\partial \widetilde{{w}^{*}}}{\partial x}& \frac{\partial \widetilde{{w}^{*}}}{\partial y}& \frac{\partial \widetilde{{w}^{*}}}{\partial z}\end{array}\right], \widetilde{{\tau }^{*}}=\left[\begin{array}{ccc}{\widetilde{{\tau }^{*}}}_{xx}& {\widetilde{{\tau }^{*}}}_{xy}& {\widetilde{{\tau }^{*}}}_{xz}\\ {\widetilde{{\tau }^{*}}}_{yx}& {\widetilde{{\tau }^{*}}}_{yy}& {\widetilde{{\tau }^{*}}}_{yz}\\ {\widetilde{{\tau }^{*}}}_{zx}& {\widetilde{{\tau }^{*}}}_{zy}& {\widetilde{{\tau }^{*}}}_{zz}\end{array}\right].$$

Now transform form of $\widetilde{{\tau }^{*}}$ is

4 $$\begin{aligned} & \widetilde{{\tau ^{*} }}_{{xx}} = - P + 2\mu \frac{{\partial \widetilde{{u^{*} }}}}{{\partial x}},\widetilde{{\tau ^{*} }}_{{xy}} = \frac{{\partial \widetilde{{u^{*} }}}}{{\partial y}} + \frac{{\partial \widetilde{{v^{*} }}}}{{\partial x}},\widetilde{{\tau ^{*} }}_{{xz}} = \frac{{\partial \widetilde{{u^{*} }}}}{{\partial z}} + \frac{{\partial \widetilde{{w^{*} }}}}{{\partial x}}, \\ & \widetilde{{\tau ^{*} }}_{{yx}} = \widetilde{{\tau ^{*} }}_{{xy}} ,\widetilde{{\tau ^{*} }}_{{yy}} = - P + 2\mu \frac{{\partial \widetilde{{v^{*} }}}}{{\partial y}},\widetilde{{\tau ^{*} }}_{{yz}} = \frac{{\partial \widetilde{{v^{*} }}}}{{\partial z}} + \frac{{\partial \widetilde{{w^{*} }}}}{{\partial y}}, \\ & \widetilde{{\tau ^{*} }}_{{zx}} = \widetilde{{\tau ^{*} }}_{{xz}} ,\widetilde{{\tau ^{*} }}_{{zy}} = \widetilde{{\tau ^{*} }}_{{yz}} ,\widetilde{{\tau ^{*} }}_{{zz}} = - P + 2\mu \frac{{\partial \widetilde{{w^{*} }}}}{{\partial z}}. \\ \end{aligned}$$

The problem that develops as a result of the linked system of PDEs is^43,44^

5 $$\frac{\partial \widetilde{{u}^{*}}}{\partial x}+\frac{\partial \widetilde{{v}^{*}}}{\partial y}+\frac{\partial \widetilde{{w}^{*}}}{\partial z}=0,$$

6 $$\widetilde{{u}^{*}}\frac{\partial \widetilde{{u}^{*}}}{\partial x}+\widetilde{{v}^{*}}\frac{\partial \widetilde{{u}^{*}}}{\partial y}+\widetilde{{w}^{*}}\frac{\partial \widetilde{{u}^{*}}}{\partial z}={v}_{nf}\frac{{\partial }^{2}\widetilde{{u}^{*}}}{\partial {z}^{2}}-\frac{{\sigma }_{nf}}{{\rho }_{nf}}{B}^{2}\widetilde{{u}^{*}},$$

7 $$\widetilde{{u}^{*}}\frac{\partial \widetilde{{v}^{*}}}{\partial x}+\widetilde{{v}^{*}}\frac{\partial \widetilde{{v}^{*}}}{\partial y}+\widetilde{{w}^{*}}\frac{\partial \widetilde{{v}^{*}}}{\partial z}={v}_{nf}\frac{{\partial }^{2}\widetilde{{v}^{*}}}{\partial {z}^{2}}-\frac{{\sigma }_{nf}}{{\rho }_{nf}}{B}^{2}\widetilde{{v}^{*}},$$

8 $$\widetilde{{u}^{*}}\frac{\partial T}{\partial x}+\widetilde{{v}^{*}}\frac{\partial T}{\partial y}+\widetilde{{w}^{*}}\frac{\partial T}{\partial z}={\alpha }_{nf}\frac{{\partial }^{2}T}{\partial {z}^{2}}+\frac{{\mu }_{nf}}{{\left(\rho {C}_{P}\right)}_{nf}}{\left(\frac{\partial \widetilde{{u}^{*}}}{\partial y}\right)}^{2}+\frac{Q}{{\left(\rho {C}_{P}\right)}_{nf}}\left(T-{T}_{\infty }\right)+\frac{16{\delta }^{*}{{T}_{\infty }}^{3}}{{3{K}^{*}\left({\rho C}_{P}\right)}_{nf}}\left(\frac{{\partial }^{2}T}{\partial {z}^{2}}\right).$$

Developing boundary conditions

9 $$\begin{aligned} & \widetilde{{u^{*} }} = U_{w} \left( x \right) = ax,T = T_{w} ,\widetilde{{v^{*} }} = V_{w} (y) = by,\widetilde{{w^{*} }} = 0:z = 0, \\ & \widetilde{{u^{*} }} \to 0,{\text{ }}\widetilde{{v^{*} }} \to 0,T \to T_{\infty } :z \to \infty . \\ \end{aligned}$$

The density, viscosity and thermal conductivity ratio of nanofluids are described as follows^43^

10 $$\begin{aligned} & \rho _{{nf}} = \left( {1 - \varphi } \right)\rho _{f} + \varphi \rho _{s} ,\mu _{{nf}} = \mu _{f} \left( {1 + B^{1} \varphi + B^{2} \varphi ^{2} } \right), \\ & \frac{{k_{{nf}} }}{{k_{f} }} = \frac{{k_{s} + \left( {m - 1} \right)k_{f} + (m - 1)(k_{s} - k_{f} )\varphi }}{{k_{s} + \left( {m - 1} \right)k_{f} - (k_{s} - k_{f} )\varphi }}. \\ \end{aligned}$$

Similarity parameters are described as follows^43^

11 $$\widetilde{{u}^{*}}=ax{f}^{\prime}\left(\eta \right), \widetilde{{v}^{*}}=by{g}^{\prime}\left(\eta \right), \widetilde{{w}^{*}}=-\sqrt{a{v}_{f}}\left(f\left(\eta \right)+cg\left(\eta \right)\right), \eta =\sqrt{\frac{a}{{v}_{f}}}z,\uptheta \left(\upeta \right)=\frac{{T-T}_{\infty }}{{T}_{w}-{T}_{\infty }}, c=\frac{b}{a}.$$

Illustrations of ordinary differential equations in the dimensionless

12 $$\varepsilon _{1} f^{\prime\prime\prime} + \left( {f + ag} \right)f^{\prime\prime} - \left( {f^{\prime}} \right)^{2} - M\varepsilon _{3} f^{\prime} = 0,$$

13 $$\varepsilon _{1} g^{\prime\prime\prime} + \left( {f + ag} \right)g^{\prime\prime} - a\left( {g^{\prime}} \right)^{2} - M\varepsilon _{3} g^{\prime} = 0,$$

14 $$(\varepsilon _{2} + {\text{Rd}})\uptheta ^{\prime\prime} + Pr\left( {f + ag} \right)\theta ^{\prime} + Pr\lambda \theta + PrEc(f^{\prime\prime})^{2} = 0.$$

The following lists the relationships based on nanoparticles. Nanoparticle characteristics are listed in Table 1 and their forms are described in Table 2.

**Table 1:** Copper and titanium thermal properties in the base liquid^43^.

Nanoparticles/base fluid	Cu	TiO_2_	H_2_O
$\rho$	8933	4250	997.1
${C}_{P}$	385	686.2	4179
$k$	401	8.9538	0.613
$\sigma$	59.6	0.125	5.5

**Table 2:** Size and shape relationships for nanoparticles^43^.

Shape of nanoparticles	Shape factors (m)	${B}^{1}$	${B}^{2}$
Platelet	5.72	37.1	612.6
Cylinder	4.82	13.5	904.4
Brick	3.72	1.9	471.4
Sphere	3.0	2.5	6.5
Blade	8.26	14.6	123.3

Boundary circumstances refers to

15 $$\begin{aligned} & f\left( 0 \right) = 0,f\prime \left( 0 \right) = 1,g\left( 0 \right) = 0,g\prime \left( 0 \right) = a,\theta \left( 0 \right) = 1,:\upeta = 0, \\ & f\prime \left( \infty \right) \to 0,g\prime \left( \infty \right) \to 0,\theta (\infty ) \to o:\upeta \to \infty . \\ \end{aligned}$$

Parameters are defined as^43,44^

16 $$M=\frac{{B}_{0}^{2}{\sigma }_{hnf}}{{\rho }_{hnf}a},Ec=\frac{{U}^{2}}{{C}_{P}\left({T}_{s}-T\right)}, Pr=\frac{\nu }{{\alpha }_{hnf}}, \lambda =\frac{Q}{a{({\rho C}_{P})}_{hnf}}, Rd=\frac{16{\delta }^{*}{{T}_{\infty }}^{3}}{{3{K}^{*}\left(k\right)}_{f}} and a=\frac{b}{c}.$$

Now ${\varepsilon }_{1}, {\varepsilon }_{2}$ and ${\varepsilon }_{3}$ are constants which are defined as^42^

$${\varepsilon }_{1}=\frac{(1+{B}^{1}\varphi +{B}^{2}{\varphi }^{2})}{(1-\varphi +\varphi \frac{{\rho }_{s}}{{\rho }_{f}})},{\varepsilon }_{2}=\frac{\frac{{K}_{hnf}}{K}}{1-\varphi +\varphi \frac{{(\rho {C}_{P})}_{s}}{{(\rho {C}_{P})}_{f}}}, {\varepsilon }_{3}=\frac{(1-\varphi +\varphi \frac{{\sigma }_{s}}{{\sigma }_{f}})}{(1-\varphi +\varphi \frac{{\rho }_{s}}{{\rho }_{f}})} .$$

Expressions without dimensions are provided for the local Nusselt number and the skin-friction factor, accordingly.

17 $$\begin{aligned} & C_{{fx}} = \frac{{2\mu _{{hnf}} \left( {\frac{{\partial u}}{{\partial z}}} \right)_{{z = 0}} }}{{\rho _{{hnf}} U^{2} }} = Re^{{ - \frac{1}{2}}} \left( {1 + B^{1} \varphi + B^{2} \varphi } \right)f^{{\prime \prime }} \left( 0 \right), \\ & C_{{fy}} = \frac{{2\mu _{{hnf}} \left( {\frac{{\partial v}}{{\partial z}}} \right)_{{z = 0}} }}{{\rho _{{hnf}} U^{2} }} = Re^{{ - \frac{1}{2}}} \left( {1 + B^{1} \varphi + B^{2} \varphi ^{2} } \right)g^{{\prime \prime }} \left( 0 \right), \\ & Nu = \frac{{ - xK_{{hnf}} \left( {\frac{{\partial T}}{{\partial z}}} \right)_{{z = 0}} }}{{T_{s} - T_{0} }} = Re^{{1/2}} \frac{{K_{{hnf}} }}{K}\uptheta \prime \left( 0 \right). \\ \end{aligned}$$

### Exploration of entropy generation

The definition of entropy generation in the mathematical model is^45,46^

18 $${E}_{G}=\frac{{k}_{f}}{{T}_{\infty }^{2}}\left[\frac{{k}_{hnf}}{{k}_{f}}+\frac{{{16\delta }^{*}T}_{\infty }^{3}}{3{k}_{f}{k}^{*}}\right] {\left(\frac{\partial T}{\partial z}\right)}^{2}+\frac{{\mu }_{hnf}}{{T}_{\infty }}{\left(\frac{\partial \widetilde{u}}{\partial z}\right)}^{2}+\frac{{\sigma }_{hnf}}{{T}_{\infty }}\left(\widetilde{u}+\widetilde{v}\right){B}^{2}.$$

The following gives a definition of dimensionless entropy creation.

19 $$NG=\frac{{T}_{\infty }^{2}{a}^{2}{E}_{G}}{{k}_{f}{\left({T}_{w}-{T}_{\infty }\right)}^{2}}.$$

Using Eq. (19), we were able to obtain the entropy equation's dimensionless form.

20 $$NG = Re\left( {\frac{{k_{{hnf}} }}{{k_{f} }} + R_{d} } \right)\theta ^{{\prime 2}} + B_{r} Re(f^{\prime\prime})^{2} + MEcPrRe\frac{1}{A}\left( {f^{{\prime 2}} + g^{{\prime 2}} } \right).$$

here

21 $$Re=\frac{{a}^{2}U}{{\nu }_{f}x}, {B}_{r}=\frac{{{\mu }_{nf}U}^{2}}{{k}_{f}({T}_{w}-{T}_{\infty })}, A=\frac{{T}_{w}-{T}_{\infty }}{{T}_{\infty }}.$$

## Method for optimal homotopy analysis

For effectiveness, the transition from the partial differential equation framework to the ordinary differential equation platform has been crucial. The generated flowing simulation (ODEs) integrating BCs is fixed employing the OHAM method. The ODE structure that occurs inside boundary constraints (BCs) is paired and irregular. Applying the optimum homotopy analysis method, we attempt to develop converging homotopy solutions^23^. The necessary initial estimates for homotopic resolutions as well as the auxiliary linear machinists are presented as

22 $${f}_{0}\left(\eta \right)=1-\mathit{exp}\left(-\eta \right),$$

23 $${g}_{0}\left(\eta \right)=a-aexp(-\eta ),$$

24 $${\theta }_{0}\left(\eta \right)={\text{exp}}\left(-\eta \right).$$

25 $$\left.{\mathcal{L}}_{f}=\frac{{d}^{3}f}{d{\eta }^{3}}-\frac{df}{d\eta }, {\mathcal{L}}_{g}=\frac{{d}^{3}g}{d{\eta }^{3}}-\frac{dg}{d\eta },{\mathcal{L}}_{\theta }=\frac{{d}^{2}\theta }{d{\eta }^{2}}-\theta .\right\}$$

The previous linear algorithms satisfy the following requirements:

26 $${\mathcal{L}}_{f}\left\{{z}_{1}^{*}+{z}_{2}^{*}exp\left(\eta \right)+{z}_{3}^{*}{\text{exp}}\left(-\eta \right)\right\}=0,$$

27 $${\mathcal{L}}_{g}\left\{{z}_{4}^{*}+{z}_{5}^{*}exp\left(\eta \right)+{z}_{6}^{*}{\text{exp}}\left(-\eta \right)\right\}=0,$$

28 $${\mathcal{L}}_{\theta }\left\{{z}_{7}^{*}exp\left(\eta \right)+{z}_{8}^{*}{\text{exp}}\left(-\eta \right)\right\}=0.$$

The aforementioned coefficients ${z}_{j}^{*}=(1-8)$, may be discovered engaging BC (boundary conditions).

### Explanations regarding graphical outcomes

The role that heat production, radiation heat, energy dissipation, and the magnetic field each play in a 3-D, 3-D thermal energy travelling simulation inside a Newtonian liquid. There are several forms of nanostructures in the basic liquid identified as water. Utilizing computational techniques, this thermal power transfer phenomena is being researched. The flowing paradigm is explained below, and a diagram showing the relationship among velocity, thermal, and other features.

### Evaluate the effect of Copper and titanium dioxide on velocity domain

**Figure 2:**
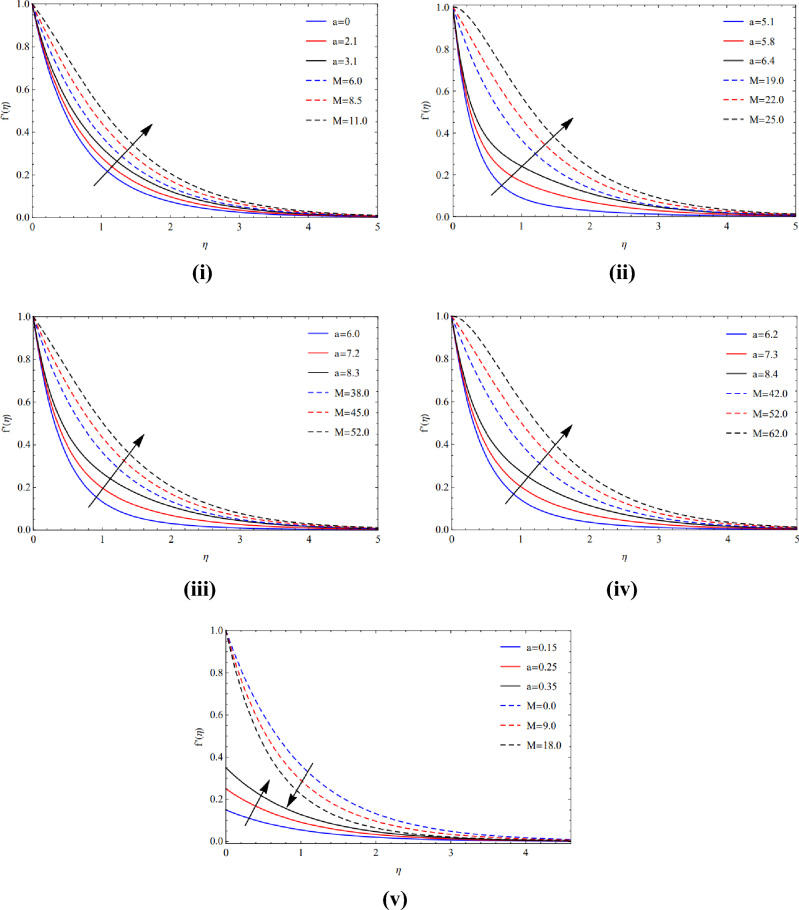
(**i**) Pose upon blade of $a$ and $M$. (**ii**) Pose upon brick of $a$ and $M$. (**iii**) Pose upon cylinder of $a$ and $M$. (**iv**) Pose upon platelet of $a$ and $M$. (**v**) Pose upon sphere of $a$ and $M$.

As contrasted to the magnetic arena, extending proportion quantity, and volume percentage with respect to various types of tiny particles, Cu and TiO_2_ tiny particles are maintained in terms of moving phenomenon. The significance of the magnetic amount $(M)$ and the elasticity proportion value $(\alpha )$ on the movement of copper tiny particles across all of their varieties. Figure 2(i)–(iv) illustrate how the motion rises as both the expanding ratio numeral $(\alpha )$ and the magnetic quantity $(M)$ raise because of Lorentz forces. In the existence of a sphere, the magnetic value and ratio factor have a consequence on the velocity sector, as seen in Figure 2(v). It is evident that when the magnetic size is increased, the motion slows down due to the Lorentz forces, however the movement in ratio numbers is the reverse of that of the magnetic digits.

**Figure 3:**
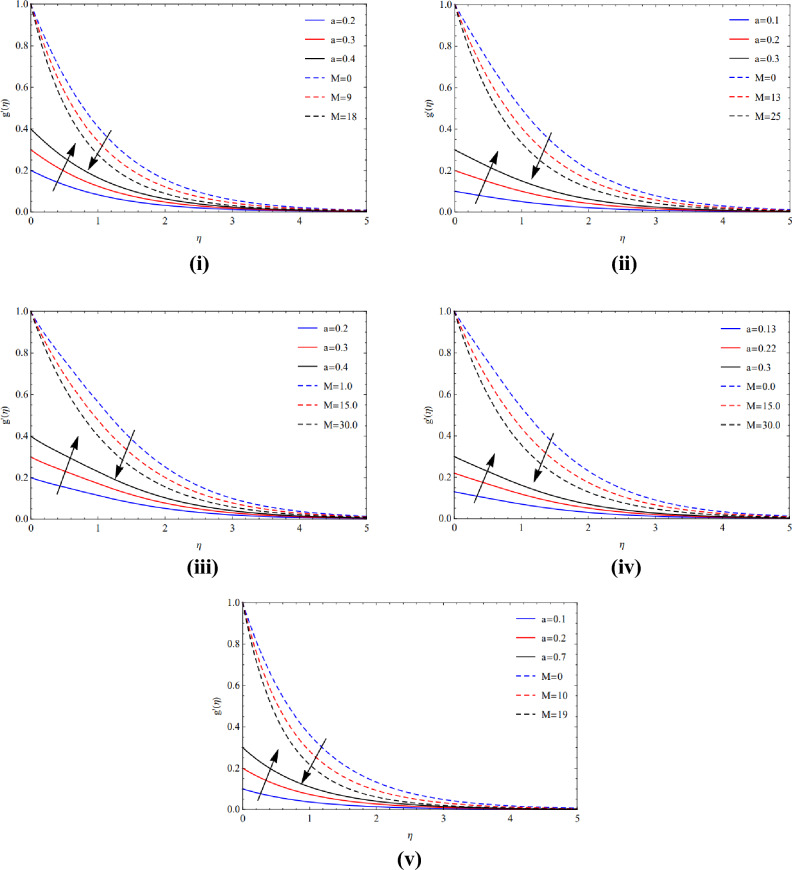
(**i**) Posture of $a$ and $M$ along Blade. (**ii**) Posture of $a$ and $M$ along Brick. (**iii**) Posture of $a$ and $M$ along cylinder. (**iv**) Posture of $a$ and $M$ along platelets. (**v**) Posture of $a$ and $M$ along sphere.

Figure 3(i)–(v) show that circulation slows down as the magnetic strength enhances while the expanded region grows for the various Cu and TiO_2_ tinyparticle varieties owing to the Lorentz force.

**Figure 4:**
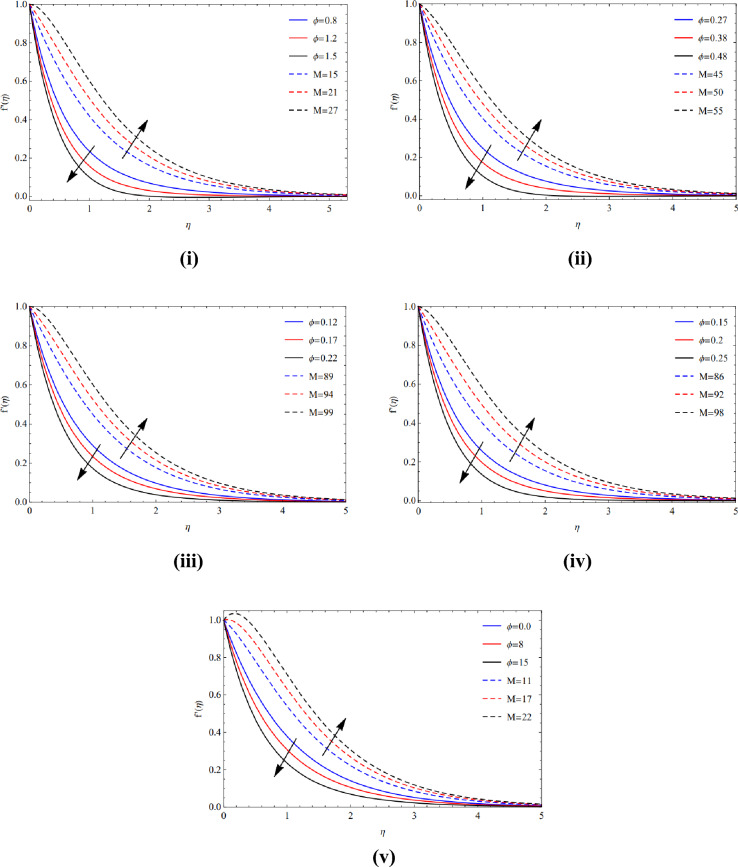
(**i**) Viewpoint towards blade of $\phi$ and $M$. (**ii**) Viewpoint towards brick of $\phi$ and $M$. (**iii**) Viewpoint towards cylinder of $\phi$ and $M$. (**iv**) Viewpoint towards platelets of $\phi$ and $M$. (**v**) Viewpoint towards sphere of $\phi$ and $M$.

The rapid growth of copper tiny materials in the existence of different forms of nanoparticles is caused by a great magnetic amount $(M)$ and the extraordinary volume friction $(\varphi )$ created via poor velocity, as seen in Fig. 4(i)–(v). Physically, the Lorentz effect is encouraged by the magnetic quantity.

**Figure 5:**
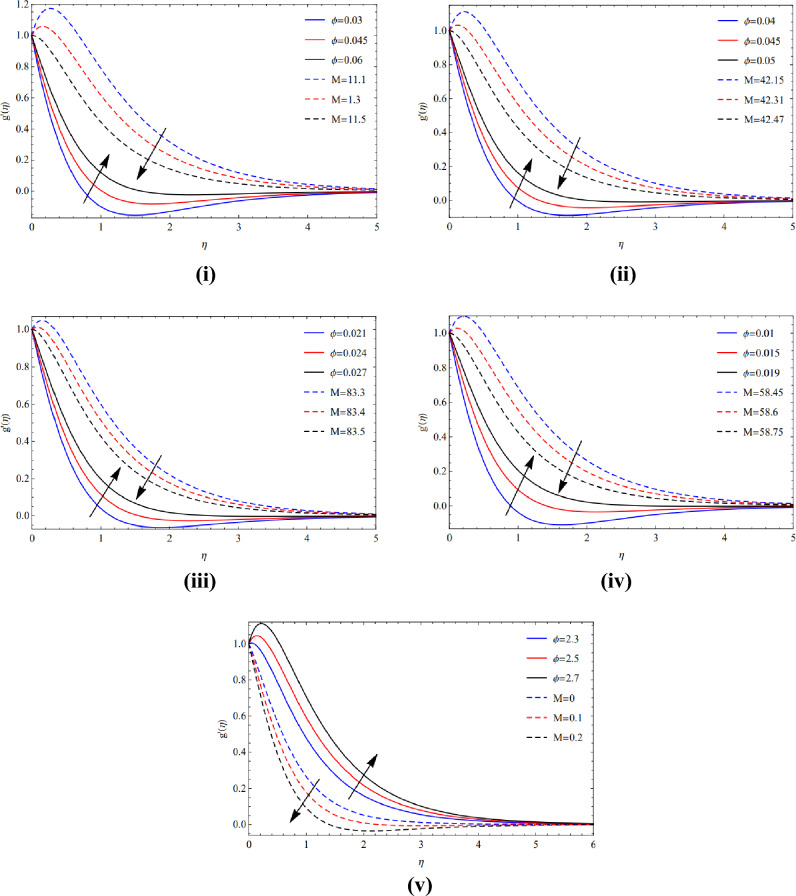
(**i**) Actions of $\phi$ and $M$ over blade. (**ii**) Actions of $\phi$ and $M$ over brick. (**iii**) Actions of $\phi$ and $M$ over cylinder. (**iv**) Actions of $\phi$ and $M$ over platelets. (**v**) Actions of $\phi$ and $M$ over sphere.

Utilizing excessive amounts of volume friction $(\varphi )$ and magnetic strength $(M)$, which cause fragile velocities, Fig. 5(i)–(v) examine the extraordinarily fast speed of copper elements in the occurrence of several kinds of nanomaterials.

### Evaluate the effects of Copper and titanium dioxide on the temperature domain

**Figure 6:**
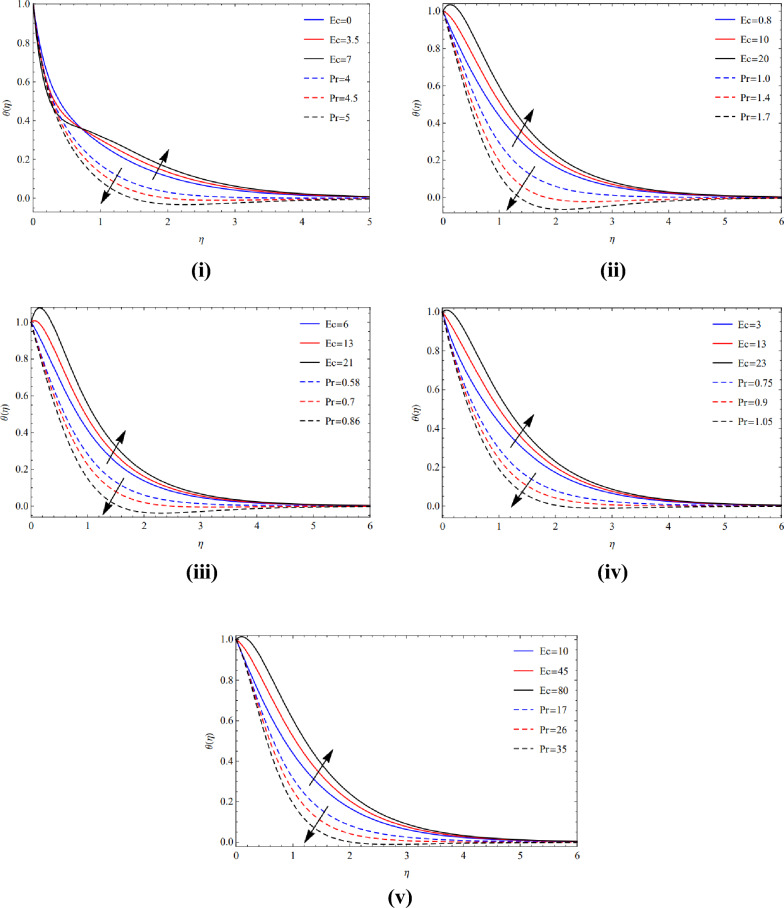
(**i**) Activity of $Ec$ and $Pr$ along blade. (**ii**) Activity of $Ec$ and $Pr$ along brick. (**iii**) Activity of $Ec$ and $Pr$ along cylinder. (**iv**) Activity of $Ec$ and $Pr$ along platelets. (**v**) Activity of $Ec$ and $Pr$ along sphere.

**Figure 7:**
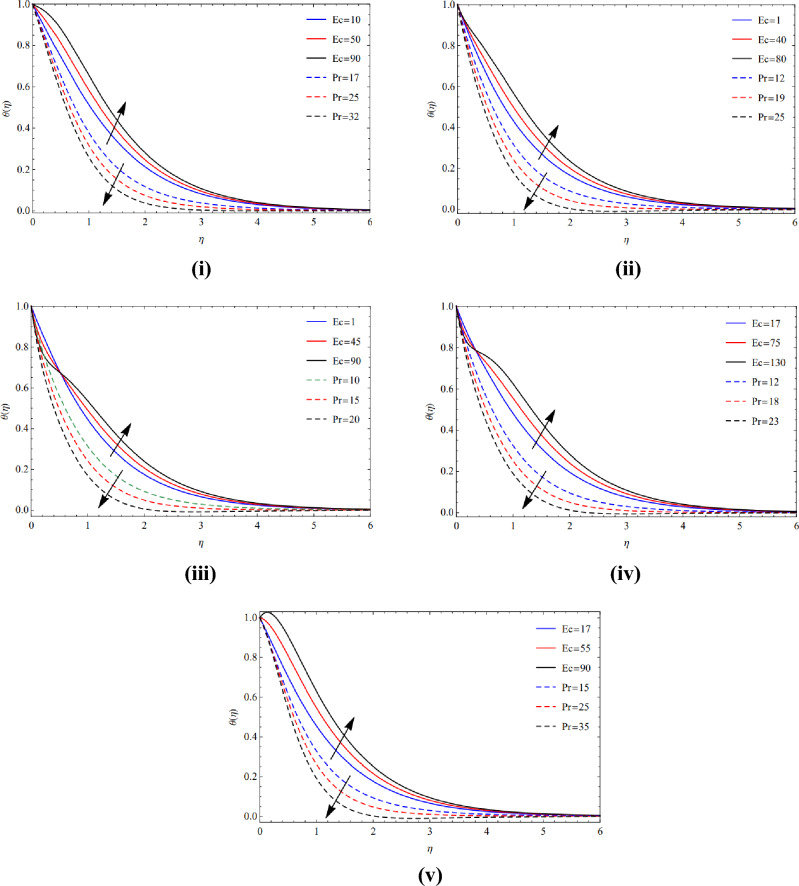
(**i**) Nature of $Ec$ and $Pr$ on blade. (**ii**) Nature of $Ec$ and $Pr$ on brick. (**iii**) Nature of $Ec$ and $Pr$ on cylinder. (**iv**) Nature of $Ec$ and $Pr$ on platelets. (**v**) Nature of $Ec$ and $Pr$ on sphere.

The Prandtl value and Eckert coefficients across the temperature region are analyzing using Figs. 6(i)–(v) and 7(i)–(v), correspondingly. These figures were generated to represent the assessment of thermal power in the existence of distinct forms and their consequences on (Cu) and TiO_2_ tiny materials. The rising value of Prandtl and Eckert computation, which generate significant heat electricity, serves as evidence that the energy from temperature is lowered in all sorts of nanoscale. Physically, decreased temperature results from thermal diffusion since the Prandtl ratio is a fundamental component of it. It is also shown that there is a clear correlation between thick dissipation and warmth energy. Eckert levels are reduced as a result of this straight link, little heat energy is released.

**Figure 8:**
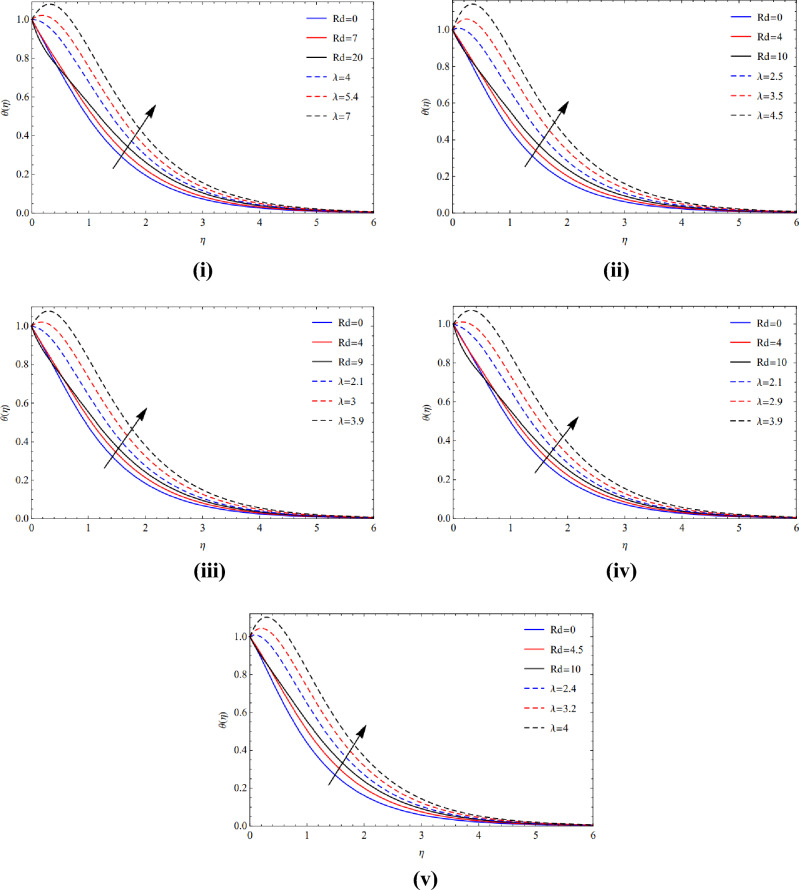
(**i**) Direction of $Rd$ and $\lambda$ on blade. (**ii**) Direction of $Rd$ and $\lambda$ on brick. (**iii**) Direction of $Rd$ and $\lambda$ on cylinder. (**iv**) Direction of $Rd$ and $\lambda$ on platelets. (**v**) Direction of $Rd$ and $\lambda$ on sphere.

**Figure 9:**
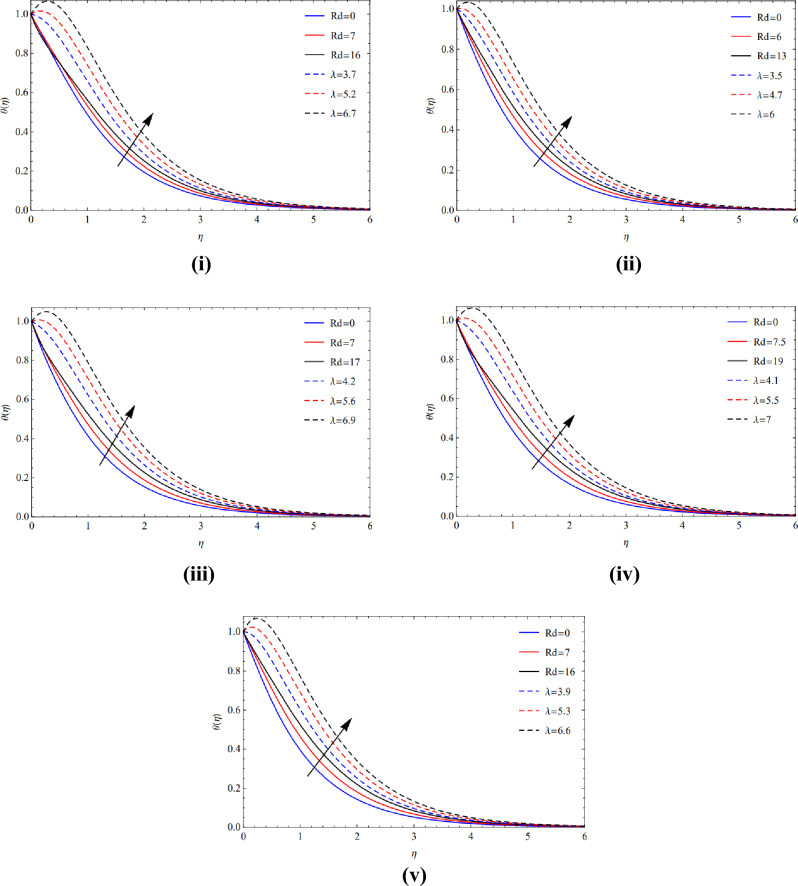
(**i**) Credit of $Rd$ and $\lambda$ at blade. (**ii**) Credit of $Rd$ and $\lambda$ at brick. (**iii**) Credit of $Rd$ and $\lambda$ at cylinder. (**iv**) Credit of $Rd$ and $\lambda$ at platelets. (**v**) Credit of $Rd$ and $\lambda$ at sphere.

Figures 8(i)–(v) and 9(i)–(v) demonstrate the notable heat production that comes from the inclusion of copper and titanium tiny particles of multiple sizes with strong radiant energy and warmth absorption indices. As thermal radiant transference of heat is closely correlated with temperature, therefore temperature scenario increases.

### Evaluate the effects of entropy generation on different parameter

**Figure 10:**
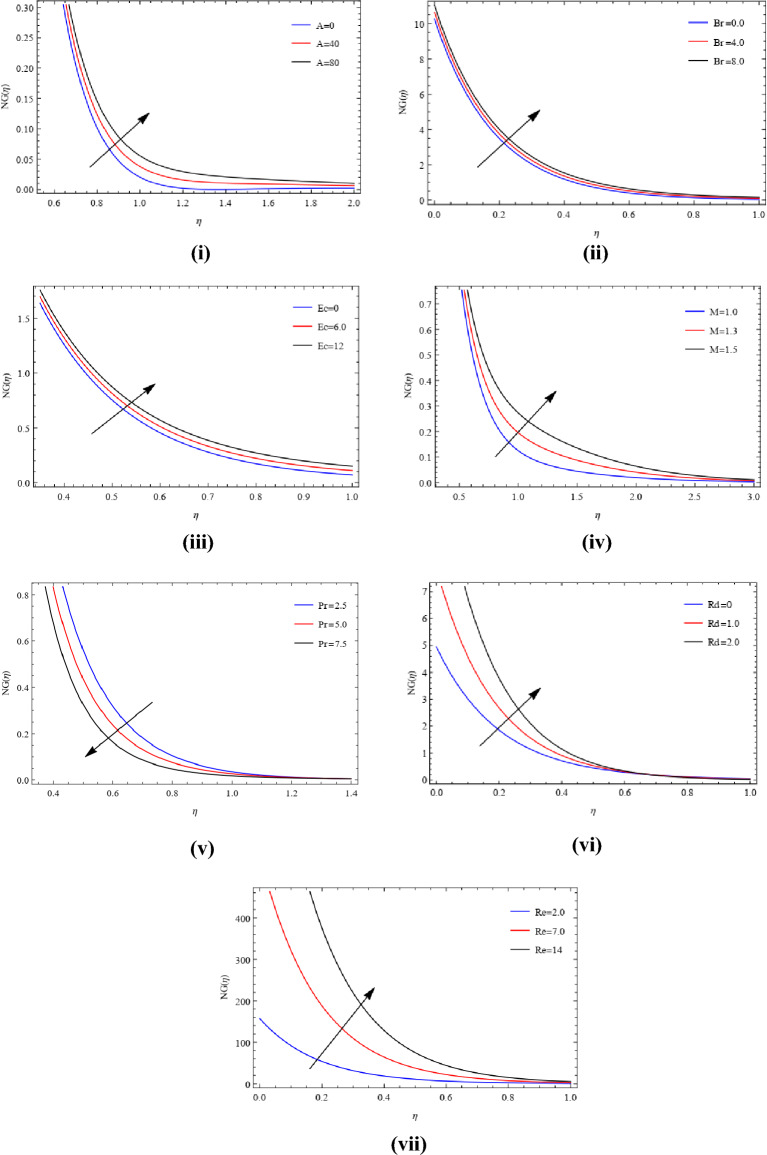
(**i**) Development of $A$ on $NG$. (**ii**) Development of $Br$ on $NG$. (**iii**) Development of $Ec$ on $NG$. (**iv**) Development of $M$ on $NG$. (**v**) Development of $Pr$ on $NG$. (**vi**) Development of $Rd$ on $NG$. (**vii**) Development of $Re$ on $NG$.

The variations in $NG$ resulting from the temperature difference A can be seen in Fig. 10(i). Here, we can see an increasing influence. The result of $Br$ on the $NG$ entropy can be seen in Fig. 10(ii). The measure of disorder increases in this situation, unlike what was previously estimated for greater Brinkman numbers. $Br$ is what creates the heat in the area where the fluid is moving. Entropy increases when there is more heat coming from inside the wall. Because of $NG$ which is improved due to lower heat conductivity caused by a larger Brinkman number. The connection across $Ec$ and $NG$ is demonstrated in Fig. 10(iii). This figure illustrates that when $Ec$ increases, the process of creating entropy speeds up. Figure 10(iv) illustrates the impact of $M$ on $NG$. The rate at which entropy is produced increases when we have higher estimates of the magnetic parameter $M$. A stronger magnetic effect makes the Lorentz force stronger, which increases the temperature by making it harder for thin film fluid to move. As shown in Fig. 10(iv), this makes the wall better at transferring heat. Figure 10(v) illustrates the directly proportional between entropy and Prandtl number. It is observed that decrease in entropy and the increase in the $Pr$ number within the flow field. The relationship between entropy and the variable $Rd$ is depicted in Fig. 10(vi). The Reynolds number has been used in Fig. 10(vii) to demonstrate how the entropy profile changes. $NG$ gets bigger when Re goes up. In simpler terms, when the Reynolds number is higher, the force from the movement of the fluid becomes stronger compared to the force caused by its stickiness. Consequently, this disturbance causes the fluid to move in a less uniform manner, ultimately resulting in increased system disorder. As a result, heat transfer makes entropy increase.

## Conclusions

The OHAM approach has been utilized to handle the issue of thermal flux in Newtonian liquids, employing a disappearing three-dimensional feature that includes nanomaterials in the dimensions of different forms mentioned in Table 2. In future we will study the hybrid nanoparticles and entropy generation using parallel plates, rotating disk and stretching cylinder. The following is an outline of the main findings of the research.

- Magnetic quantity $(M)$ levels result in significant velocities and an upsurge in movement.
- The deformation proportion $(\alpha )$ is large, wall momentum disperses quicker while the magnetic size $(M)$ is excessive, and it disperses more sluggishly.
- As volumetric frictional $(\varphi )$ increases, fragile velocity is produced.
- Heating energy is diminished as a result of the great Prandtl ratio $(Pr)$.
- Strong Eckert quantity $(Ec)$ results in greatest warmth generation.
- Radiant heat $(Rd)$ and energy absorption $(\lambda )$ have stimulus on thermal distribution.
- The entropy $(NG)$ has been discovered to rise when the magnetic parameter $(M),$ porosity factor $(A)$, eckert number $(Ec)$, thermal radiation $(Rd)$, reyloand number $(Re)$ and brinkman number $(Br)$ rise. When increase the value of prandtl the entropy decrease.

## Data Availability

The datasets used and/or analyzed during the current study available from the corresponding author on reasonable request.

## Abbreviations

$\widetilde{{u}^{*}},\widetilde{{v}^{*}},\widetilde{{w}^{*}}$: Velocity components (ms^−1^)
$${\nu }_{nf}$$: Kinematic viscosity (m^2^ s^−1^)
$${\sigma }_{nf}$$: Electricity conductivity (Ω^−1^ m^−1^)
$${\rho }_{nf}$$: Density (kg m^−3^)
$B$: Magnetic field strength (Ω^1/2^ m^−1^ s^−1/2^ kg^1/2^)
$\frac{{K}_{nf}}{K}$: Thermal conductivity (Wm^−1^ K^−1^)
$${\rho C}_{P}$$: Heat capacitance (J m^−3^ K^−1^)
$$\varphi$$: Volume friction
$m$: Shape factor nanoparticles
$$a$$: Stretching velocity (s^−1^)
$Rd$: Thermal Radiation Parameter
$$Q$$: Heat generation
${\delta }^{*}$: Stefan Boltzmann constant (W m^−2^ K^−4^)
${B}^{1},{B}^{2}$: Viscosity enhancement coefficient
$$Pr$$: Prandtl number
$$M$$: Magnetic parameter
$$N{u}^{*}$$: Nusselt number
$$Ec$$: Eckert number
$${C}_{fx},{C}_{fy}$$: Skin friction
${k}_{s}$: Thermal conductivity nanoparticle (W m^−1^ K^−1^)
$${T}_{s}$$: Reference temperature (K)
$$T$$: Temperature nanofluid (K)
$$U$$: Velocity (m s^−1^)
$\lambda$: Heat generation parameter
$${T}_{\infty }$$: Ambient temperature (K)
${K}^{*}$: Mean absorption coefficient (m^−1^)
